# A randomised controlled trial of the *Learning Skills Together* (*LST*) intervention to improve dementia family caregivers’ self-efficacy with complex care

**DOI:** 10.1186/s13063-024-08204-8

**Published:** 2024-06-08

**Authors:** Kylie Meyer, Kyungmi Lee, Sutthinee Thorngthip, Patricia Burant, Megan Lippe, Daria Neidre, Carole White, Rocio Norman, Byeong Yeob Choi, Crystal M. Glover, Janice Bell, Kenneth Hepburn

**Affiliations:** 1 Frances Payne Bolton School of Nursing, Case Western Reserve University, Cleveland, OH 44106 USA; 2 School of Nursing, University of Texas Health Science Center at San Antonio, San Antonio, TX USA; 3 Biggs Institute on Alzheimer’s Disease and Related Dementias, University of Texas Health Sciences at San Antonio, San Antonio, TX USA; 4 School of Health Professions, University of Texas Health Science Center at San Antonio, San Antonio, TX USA; 5 Department of Psychiatry and Behavioral Sciences, Division of Behavioral Sciences, RUSH Medical College, Chicago, IL USA; 6 Department of Neurological Sciences, RUSH Medical College, Chicago, IL USA; 7 Rush Alzheimer’s Disease Center, RUSH Medical College, Chicago, IL USA; 8 Betty Irene Moore School of Nursing, University of California Davis, Sacramento, CA USA; 9 Nell Hodgson Woodruff School of Nursing, Emory University, Atlanta, GA USA

**Keywords:** Alzheimer’s, Dementia, Family caregiving, Non-pharmacological intervention, Randomized controlled trial

## Abstract

**Background:**

Nearly two-thirds of family caregivers of persons living with Alzheimer’s disease or related dementias (AD/ADRD) provide complex care, including medical care. Family caregivers typically receive little to no training on how to provide this care. Furthermore, family caregivers simultaneously grapple with the presence of behavioral and psychological symptoms of dementia (BPSD), diminished communication abilities, and comorbidities such as diabetes. We developed *Learning Skills Together* (*LST*), a 6-week digitally delivered psychoeducational program, to facilitate family caregiver abilities to administer complex care tasks. The goal of the present study is to test the efficacy of *LST* and to reduce adverse outcomes associated with caregiving, such as depressive symptomology and negative appraisal of BPSD.

**Methods:**

To test the efficacy of *LST*, we will conduct a two-arm single-site randomized controlled trial (RCT) with *N* = 200 family caregivers of persons living with AD/ADRD. Eligible family caregivers will be randomly assigned to participate in either the *LST* intervention or a structurally equivalent control condition focused on healthy living. All family caregivers will complete four surveys, including a baseline survey administered prior to randomization, a post-intervention survey, and a 3- and 6-month follow-up survey to assess change in study outcomes. Between-group comparisons of each outcome will be evaluated using generalized estimating equation models. Mediation analyses will assess family caregiver self-efficacy as the intervention’s mechanism of change in depressive symptomology and BPSD. We will also examine caregiver race, ethnicity, and gender as effect modifiers of the intervention.

**Discussion:**

*LST* findings will inform the field of AD/ADRD and caregiving regarding optimally supporting family caregivers in managing complex care tasks. If efficacious, the *LST* intervention will support family caregivers in preserving their own mental health while providing complex care.

**Trial registration:**

Clinical Trials.gov NCT05846984. This study was registered on May 6, 2023.

## Administrative information

Note: the numbers in curly brackets in this protocol refer to SPIRIT checklist item numbers. The order of the items has been modified to group similar items (see http://www.equator-network.org/reporting-guidelines/spirit-2013-statement-defining-standard-protocol-items-for-clinical-trials/).

**Table:** 

Title {1}	A randomised controlled trial of the *Learning Skills Together* (*LST*) intervention to improve dementia family caregivers’ self-efficacy with complex care
Trial registration {2a and 2b}.	This study was registered on Clinical Trials.gov on May 6, 2023 (NCT05846984)
Protocol version {3}	February 11, 2024, Version 1
Funding {4}	The study is supported by National Institute on Aging (NIA) under award number R01AG077554 (Meyer)
Author details {5a}	Kylie Meyer, PhD, MSc^1^ Kyungmi Lee, PhD, RN^1^ Sutthinee Thorngthip, BSN, RN^1^ Patricia Burant, EdD^1^ Megan Lippe, PhD, MSN, RN, FAAN^2^ Daria Neidre, PhD, MA^3^ Carole White, PhD, RN, FAAN ^2^ Rocio Norman, PhD, CCC-SLP ^3^ Byeong Yeob Choi, PhD^4^ Crystal M Glover, PhD^6å,7,8^ Janice Bell, Ph.D., MPH, MN, FAAN ^9^ Kenneth Hepburn, PhD^10^ ^1^ Frances Payne Bolton School of Nursing, Case Western Reserve University, Cleveland, OH, USA ^2^ School of Nursing, University of Texas Health Science Center at San Antonio, San Antonio, TX, USA ^3^ Biggs Institute on Alzheimer’s Disease and Related Dementias, University of Texas Health Sciences at San Antonio, San Antonio, TX, USA ^4^ School of Health Professions, University of Texas Health Science Center at San Antonio, San Antonio, TX, USA ^5^ Department of Population Health Sciences, University of Texas Health Science Center at San Antonio, San Antonio, TX, USA ^6^ Department of Psychiatry and Behavioral Sciences, Division of Behavioral Sciences, RUSH Medical College, Chicago, IL, USA ^7^ Department of Neurological Sciences, RUSH Medical College, Chicago, IL, USA ^8^ Rush Alzheimer's Disease Center, RUSH Medical College, Chicago, IL, USA ^9^ Betty Irene Moore School of Nursing, University of California Davis, Sacramento, CA, USA ^10^ Nell Hodgson Woodruff School of Nursing, Emory University, Atlanta, GA, USA *Correspondence: Kylie Meyer, Frances Payne Bolton School of Nursing, Case Western Reserve University, Cleveland, OH, 44106
Name and contact information for the trial sponsor {5b}	National Institutes of Health (NIH) 9000 Rockville Pike, Bethesda, MD 20892, USA
Role of sponsor {5c}	The National Institute on Aging was not involved in the study design; collection, management, analysis, interpretation of data, nor the writing of this report or the decision to submit the report for publication.

## Introduction

### Background and rationale {6a}

Family caregivers increasingly provide complex care tasks for persons with Alzheimer’s disease and related dementias (AD/ADRD) [1, 2]. These tasks encompass both personal care duties and medical/nursing activities, such as monitoring the side effects of new medications, communicating with the healthcare team, and preparing special diets to manage chronic conditions like diabetes and hypertension [3, 4]. The loss of the ability to self-manage co-morbidities among persons living with AD/ADRD, such as following care plans and managing follow-up care, means that family caregivers often help in the management of multiple complex conditions.

Caregivers to persons living with AD/ADRD face additional challenges than those caring for cognitively intact individuals [3, 5–8]. These caregivers must apply AD/ADRD knowledge even when managing routine personal care, such as monitoring for and preventing dysphagia during feeding assistance [9]. Caregivers to persons living with AD/ADRD must also manage behavioral and psychological symptoms of dementia (BPSD), characterized by disturbances in mood and behavior that affect nearly all persons living with AD/ADRD [10]. BPSD are positively associated with both caregiver burden and depressive symptomology and contribute to resistance to care from care recipients [11, 12]. Communication challenges add another layer of difficulty when delivering complex care to persons living with AD/ADRD, as care recipients may be unable to communicate their needs or express pain [4].

Complex care tasks performed by caregivers to persons living with AD/ADRD are vital not only to the care receiver, families, and communities but also to the long-term care system [7, 13]. Previous studies have noted that the US $600 billion estimated value of care provided by family caregivers in the U.S. outstrips costs paid to formal long-term supports and services from all sources, including Medicare and Medicaid [14]. Yet, 53% of caregivers to persons living with AD/ADRD undertake these tasks without prior training, which may contribute to increased worry about potential errors and emotional distress [3, 4, 6, 15]; furthermore, 22% of caregivers who perform complex care tasks report difficulty doing so [16].

There is a need for targeted training and support programs tailored for caregivers of persons living with AD/ADRD to better prepare them to meet their family member’s complex care needs. Existent caregiver training interventions often lack specificity to unique circumstances of caring for persons with AD/ADRD (e.g., presence of BPSD), and current interventions to support caregivers to provide complex care frequently do not incorporate evidence-based psychoeducational approaches that have shown effectiveness in AD/ADRD caregiver interventions [17, 18]. Strategies such as active learning components and problem-solving methods, known to enhance existing caregiver interventions, remain underutilized [17, 18]. A focus on self-efficacy may bolster caregivers’ ability to administer complex care tasks to their family member living with AD/ADRD.

Self-efficacy—which refers to the belief in one's ability to accomplish tasks and achieve desired outcomes [19]—plays a critical role in the mental health and well-being of caregivers [20, 21]. Higher levels of self-efficacy are associated with better outcomes and greater satisfaction in the caregiving role [20, 22–24]. Furthermore, self-efficacy can mediate the association between the perceived severity of BPSD and depression, underscoring its importance in managing complex care tasks [23, 25].

To address self-efficacy among family caregivers to persons living with AD/ADRD, we developed the *Learning Skills Training* (*LST*) program, a psychoeducational intervention to enhance caregiver self-efficacy in managing complex care tasks. The current study aims to test the efficacy of the *LST* program. Prior pilot studies indicate that *LST* is acceptable to caregivers and feasible to deliver [26, 27]. Findings from a pre- and post-test pilot trial conducted with *N* = 35 caregivers at an academic service center from 2020 to 2021 further identified improvements in caregiver self-efficacy [28]. Caregivers who participated in this pilot study completed self-administered online surveys before participating in *LST*, and twice more 4 weeks and then 8 weeks post-intervention. Statistically significant improvements in caregiver overall competence and self-efficacy with complex care were observed at both follow-up time points compared to baseline scores. The current study aims to expand on earlier feasibility findings by testing the efficacy of *LST* in a randomized controlled trial (RCT) relative to a control condition at improving caregiver mental health. Ultimately, this research aims to contribute to the development of effective interventions for caregivers to persons living with AD/ADRD, empowering them with essential skills and knowledge to reduce distress, increase self-efficacy, and provide high-quality care.

## Objectives {7}

### Primary objective

The primary objective of this study is to determine whether participation in the *LST* intervention: (1) improves caregiver self-efficacy (primary outcome) overall and related to complex care for persons living with AD/ADRD and (2) reduces depressive symptomatology and negative appraisals of BPSD (secondary outcomes) relative to a structurally equivalent control condition among family caregivers to persons living with AD/ADRD. Further, the investigators will examine whether self-efficacy functions as a mediator of secondary outcomes. If a mediating effect is found, it would support the application of self-efficacy as an intervention mechanism.

### Secondary objectives

The secondary objective of this study is to determine whether intervention effects, if found, occur equitably among caregivers according to caregiver race, ethnicity, and gender. Race/ethnicity (non-Hispanic Black, non-White Hispanic) and female gender will be tested as effect modifiers. Recent findings suggest that African American caregivers, for example, may benefit less than caregivers from other racial backgrounds in self-efficacy-focused interventions due to a potential ceiling effect [29].

#### Ancillary objectives

Given the importance of identifying the change mechanism to understand the catalyst of intervention effects, we will also conduct alternative theory testing [30]. Resourcefulness will be tested as a mediator in place of self-efficacy. “Resourcefulness” encompasses personal and social skills used to manage life challenges and has been found to mediate decreased depressive outcomes among family caregivers to persons living with AD/ADRD who participated in the Resourcefulness Training intervention [31, 32]. Quality of care provided to care recipients, such as the provision of care that is respectful and meets their needs, will also be considered a secondary outcome in addition to caregiver mental health.

### Trial design {8}

This study uses a RCT design involving two parallel groups equally allocated to the intervention and control conditions (*n* = 100 per arm; 1:1 allocation). In this study, the research team aims to determine whether the intervention condition demonstrates superiority relative to the active control condition. The investigators will oversample caregivers who identify as non-Hispanic Black/African American and non-White Hispanic caregivers to examine differences in intervention effects by race and ethnicity. These populations were selected for further study due to the heightened risk of AD/ADRD relative to non-Hispanic whites [1]; access to efficacious interventions among non-Hispanic Black/African American and non-White Hispanic families may still be more critical relative to other populations due to heightened disease prevalence.

## Methods: participants, interventions, and outcomes

### Study setting {9}

All research activities will be conducted remotely within the USA, including data collection and delivery of the intervention and control group. Participants will be asked to join Zoom-based intervention sessions.

### Eligibility criteria {10}

#### Participants

To be eligible for the study, individuals must be ages 18 years or older and a family member, including “families of choice,” of an individual living with AD/ADRD diagnosed by a physician. Consistent with prior caregiver intervention studies, caregivers must help with at least two instrumental activities of daily living (e.g., managing finances) or one activity of daily living (e.g., bathing). To ensure caregivers assist a person living with mid-stage AD/ADRD, caregivers must report a Global Deterioration Scale (GDS) rating for care recipients between 4 and 6 [33]. Participants must be able to commit to attending at least 5 of 6 synchronous Zoom sessions. Further, individuals must have reliable access to the internet and email and be able to attend synchronous sessions using the Zoom videoconferencing platform. To promote access, individuals may meet this criterion by borrowing a device and/or using a WiFi access device provided by the study team. Participants will be excluded if they are unable to read and/or speak English, participated in *Learning Skills Together* in the past, plan to place their family member in a skilled nursing facility in the next 9 months (i.e., the study duration), or were diagnosed with, started, or significantly altered their depression treatment, including starting a pharmacological therapy or beginning therapy, in the previous 3 months.

#### Interventionists

Interventionists who are eligible to deliver the intervention or control condition are registered nurses (RNs) with a Bachelor’s degree level of training, at minimum.

### Who will take informed consent? {26a}

This study will use a digital consent form. Eligible caregivers will receive an email with a unique link to the consent form, which is sent by the project manager, trained research assistants, or the principal investigator. Prior to sending this online form, key information from the consent document is reviewed verbally by these same team members. To assess their understanding, caregivers must correctly answer five true or false questions before signing the consent form. If a caregiver provides an incorrect response, the PI will call the participant to review study information and repeat true and false questions over the phone. This is done to rule out the possibility that a response was accidently mis-marked.

### Additional consent provisions for collection and use of participant data and biological specimens {26b}

Each time caregivers complete a follow-up survey, they will be reminded of key points of the consent form, such as the voluntary nature of the study and the option to skip questions if they so choose. This trial does not involve collecting biological specimens for storage.

## Interventions

### Explanation for the comparison or control condition {6b}

A structurally equivalent control condition was selected to prevent the likelihood that exposure to caregiver peers and/or the facilitator might explain differences in intervention effects, rather than intervention components designed uniquely to improve self-efficacy. As described, content is developed to build knowledge and explain information about healthy living and excludes active-learning components believed to enhance self-efficacy.

### Intervention description {11a}

#### Learning skills together intervention

*Learning Skills Together* is a 6-week psychoeducational intervention. Each week, participants are asked to join discussion group sessions of 6 to 12 caregivers over videoconference, lasting 1.5 h each. Videoconference sessions will introduce topics covered during a given week (e.g., communicating with someone living with dementia). Sessions are designed to prioritize discussion and application of information such as thorough case studies, sharing examples among participants, and group exercises. In-between sessions, participants are asked to review written and video materials in the participant workbook to achieve an in-depth understanding of topics. The workbook also contains interactive materials, including reflection exercises to support the application of information and multiple-choice “knowledge check” questions. Participants will also complete a practice exercise relevant to the subject of that week’s lesson. Practice exercise experiences will be discussed during videoconference sessions the following week. This framework is influenced by the successful models of the *Tele-Savvy* and *Building Better Caregivers* programs [34, 35]. The team selected to deliver the program over six sessions given prior evidence that this is the optimal number for interventions targeting self-efficacy [36]. Topics are listed in Table 1.

**Table 1:** Topics covered in each intervention arm

*Learning Skills Together*	Healthy living control condition
• Behavioral symptoms of dementia • Communication with a person living with AD/ADRD • Home safety • Transfers (e.g., bed to wheelchair) • Nutrition and mealtimes • Oral hygiene • Dysphagia • Incontinence • Medication management • Assessment of health conditions • Communicating with healthcare providers • Advance directives • Hospice and palliative care	• Overview of self-care • Health risks of caregiving • Healthy eating and nutrition • Physical activity • Mental health • Social well-being • Sleep hygiene • Monitoring health • Advance directives

### Control condition

The control condition focuses on healthy living for family caregivers and consists of six weekly Zoom discussion sessions. Like *LST*, sessions last 1.5 h each and will be attended by 6 to 12 caregivers. Sessions will consist of information delivery, such as explaining the importance of certain dietary choices, rather than applying information such as by using group exercises. Attendees will be asked to read in-depth information provided in their participant workbook after each session. Interactive components such as reflection exercises and knowledge checks are not incorporated into the control group workbook to reduce the possibility of contamination. Further, there is no required weekly practice exercise in the control condition. A previous trial discovered that an educational intervention promoting a healthy lifestyle for caregivers of individuals with AD/ADRD, based on the National Institute on Aging’s *Go4Life* content, did not lead to enhancements in self-efficacy, depression levels, or the assessment of BPSD [34]. Topics for the control condition are listed in Table 1.

### Criteria for discontinuing or modifying allocated interventions {11b}

Participants may choose to withdraw from the study at any time. Participants may withdraw already collected research data so long as data are not yet de-identified. Participants may also be withdrawn if they become ineligible to participate, such as if the person they care for passes away. Involuntary withdrawal may occur if it is suspected that an individual is impersonating a caregiver or if their participation in the intervention or control group programs is considered overly disruptive to other participants. If this occurs, the participant will be notified by email. We will retain study data collected to that point unless otherwise requested.

### Strategies to improve adherence to interventions {11c}

#### Interventionist adherence

Multiple strategies will be used to ensure consistent assessment of interventionist adherence. Both the intervention and control arm will be administered by a nurse interventionist holding an RN credential, thereby establishing a common training baseline for all interventionists. Further, training will be provided to all facilitators before intervention delivery, including an explanation of the background of each program, standards for the delivery of each arm, and rehearsal of delivering sessions. Training includes self-study of material, didactic training with the PI and/or project manager, and role-play of intervention delivery. Standardized materials will reinforce adherence, including intervention slides, talking points, and a facilitator guide. One videoconference session per cohort will be randomly selected for fidelity monitoring for both *LST* and the control condition. This monitoring entails interventionist self-assessment and a review of a recorded session by an observer using a standardized form. Interventionists will meet at least quarterly with the PI and project manager to discuss any issues arising during delivery.

#### Participant adherence

To support participants’ adherence to the assigned intervention, interventionists will call participants to introduce themselves and answer any questions they may have before the start of the session. Before each scheduled video conference, interventionists will send two reminder emails. After each videoconference session, participants will receive a concise summary email and a reminder about what participants should do before the next session.

### Relevant concomitant care permitted or prohibited during the trial {11d}

Relevant concomitant interventions are permitted during the trial.

#### Provisions for post-trial care {30}

There are no planned provisions for post-trial care, other than to provide written program materials to participants for whichever program into which they were not randomized. As the likelihood of harm from participation in the trial is low, there is no plan for compensation for those who suffer harm from trial participation.

### Outcomes {12}

Outcome measures will be assessed using the average change score from baseline scores until each post-intervention survey. Follow-up data collection will occur within 3 weeks post-intervention, 3 months post-intervention, and 6 months post-intervention.

### Primary outcome measures

#### Overall family caregiver self-efficacy

The primary outcome measure in this trial will be the change in caregivers’ average scores on the 8-item Caregiver Self-Efficacy Scale from baseline until each follow-up survey [37]. This measure was selected because it provides a more concise measure of self-efficacy than prior, domain-specific scales and demonstrates reliability (*α* = 0.88 to 0.89). On a scale of 1 to 10, participants are asked to rate their confidence with 8 aspects of caregiving (e.g., controlling upsetting thoughts). Scores range from 8 to 80, with higher scores indicating higher caregiver self-efficacy levels.

#### Family caregiver self-efficacy with complex care

There are no validated scales to measure caregiver self-efficacy specific to complex care. Thus, the authors generated a 16-item measure structured after the Caregiver Self-Efficacy Scale that asks caregivers to rate their confidence in handling various complex care tasks. This 16-item scale asks about how confident caregivers feel, from 0 (not at all confident) to 5 (very confident), with various complex care tasks (e.g., Managing incontinence issues) [28]. Pilot data showed high internal consistency (*α* = 0.89). Scores range from 0 to 80, with higher scores indicating higher caregiver self-efficacy levels with complex care.

While not measuring self-efficacy per se, the related construct of caregiver confidence with complex care will also be measured using the Caregiver Confidence in Sign/Symptom Management Scale (*α* = 0.91), which subscales for Knowledge of Symptoms (*α* = 0.56), Management of Cognitive Symptoms (*α* = 0.82), Management of Medical Symptoms (*α* = 0.78), and General Medical Management (*α* = 0.94) [38]. Caregivers are asked how “true” statements are regarding their (1) knowledge, (2) ability to care for, and (3) make decisions about complex care tasks, as well as their level of confidence with various tasks. Scores range from 25 to 125, with higher scores indicating higher caregiver self-efficacy levels with complex care.

### Secondary outcome measures

#### Change in family caregiver depressive symptomology

Depression will be measured with the Patient Health Questionnaire-9 (PHQ-9) [39]. The PHQ-9 demonstrates specificity and sensitivity at 74–88% and 88–91%, respectively, for major depression with a cut-off score of 10 [39, 40]. Scores range from 0 to 27, where higher scores indicate higher levels of depressive symptomology.

#### Family caregiver appraisal of behavioral symptoms of dementia

Appraisal of behavioral symptoms of dementia will be measured with the Revised Memory and Behavior Checklist (RMBC) [41]. The RMBC includes 24 items and asks about the caregiver’s appraisal of behavioral and psychological symptoms of dementia that are present in the last week (e.g., talking loudly or rapidly; *α* = 0.90). Participants may indicate whether they feel extremely bothered or upset (4), very much bothered or upset (3), moderately bothered or upset (2), a little bothered or upset (1), not at all bothered or upset (0), or did not occur in the past week (0). Scores range from 0 to 96, wherein higher scores indicate higher levels of bother (more negative appraisal of behavioral symptoms of dementia). The outcome measure will use the average change score from baseline scores until each post-intervention survey (i.e., post-intervention, 3 months post-intervention, 6 months post-intervention).

### Ancillary outcome measures

#### Change in family caregiver resourcefulness

Resourcefulness is measured using the 28-item Caregiver Resourcefulness Scale (*α* = 0.85) [42]. This scale has two factors: (1) help-seeking and (2) self-help. Caregivers are asked the frequency with which they use different strategies to manage challenges and may respond: not at all like me (0), pretty much not like me (1), a little bit not like me (2), a little bit like me (3), pretty much like much like me (4), or very much like me (5). Items are added together to create a total score. Scores range from 0 to 140, where higher scores indicate higher levels of resourcefulness.

#### Change in quality of care by caregiver

Quality of caregiving according to the caregiver will be measured with the Task Management Strategy Index (TMSI; *α* = 0.74 to 0.81) [43]. The 19-item TMSI was developed to assess caregivers’ ability to manage their family member’s functional disabilities [44]. Caregivers are asked how often they engage in strategies that support quality care. Caregivers indicate never (1), rarely (2), sometimes (3), often (4), or always (5). Scores range from 19 to 95. Higher scores indicate higher quality of caregiving.

### Participant timeline {13}

**Fig. 1:**
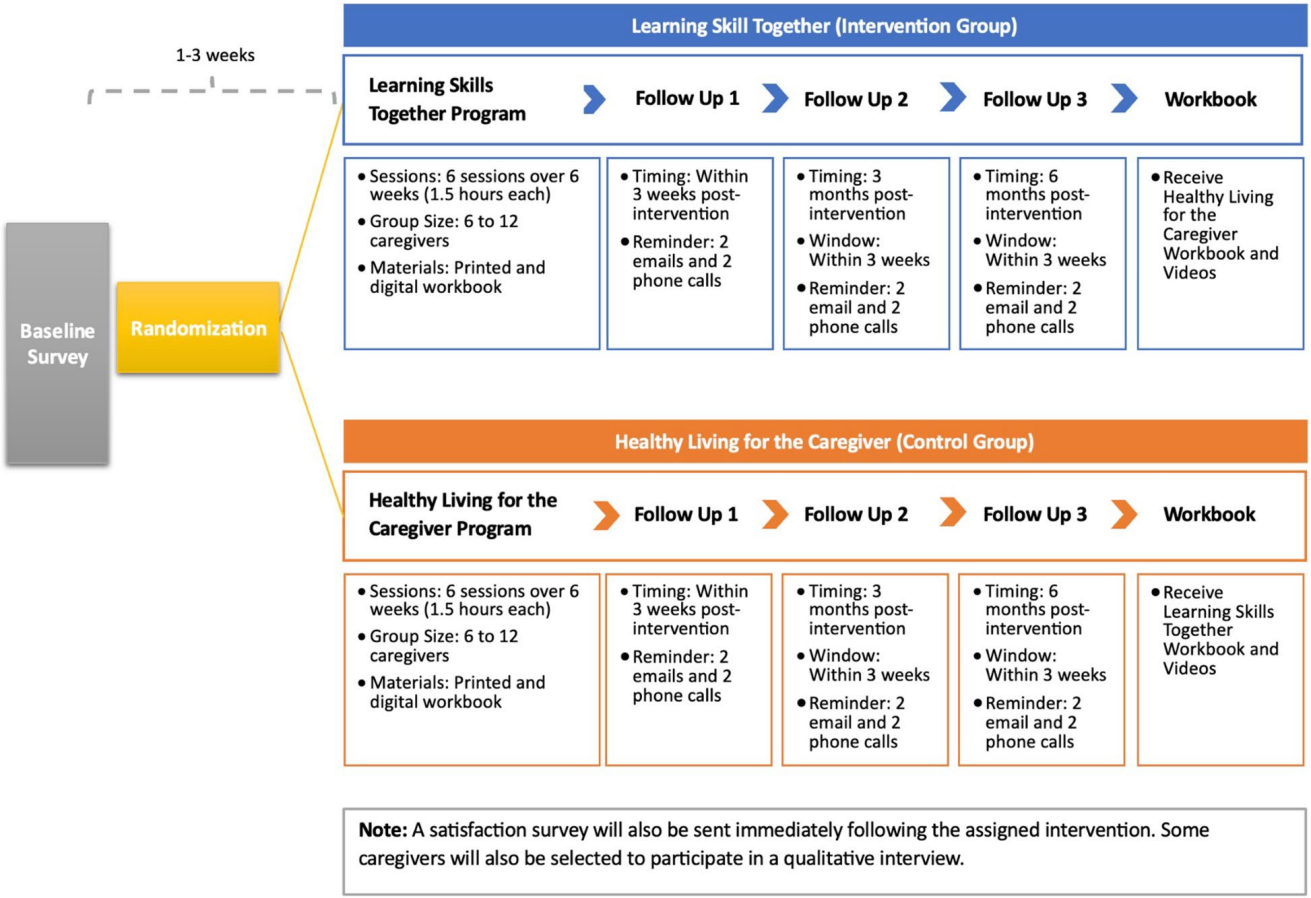
Timeline of study events for participants

Participants will complete a baseline survey up to 3 weeks before randomization. Once randomized, they will participate in either the intervention or control group condition once weekly for 6 weeks. A follow-up survey will be completed within 3 weeks post-intervention. A third and fourth follow-up survey will be administered 3 and 6 months post-intervention. Once the participant completes all study activities, they will receive the workbook materials for whichever program into which they were not randomly allocated to participate. Figure 1 illustrates this timeline.

### Sample size {14}

We will enroll a sample of *N* = 200 caregivers for randomization with a 1:1 ratio. This number is based on an effect size of *d* = 0.68 identified in a 2-month pre- and post-test pilot study for self-efficacy scores [28]. Given that control group participants may also experience some improvement from baseline, we reduced the anticipated effect size to *d* = 0.54 at 6 months. The corresponding sample size estimate is *N* = 110 to detect an effect size of *d* = 0.54 with 80% statistical power using two-sample *t*-tests with *α* = 0.05. Because using longitudinal data can increase power, we also performed power analysis using PASS v14 to calculate the detectable time-averaged effect size based on changes from baseline. A sample size of *N* = 110 can detect the time-averaged effect size of 0.42 when repeated measurements have an AR(1) covariance structure with a correlation coefficient of 0.2. We anticipate that the effect sizes at post-intervention and 3 months will be at least 0.4 and 0.5, respectively, and thus a significant intervention effect will be observed if the effect size at 6 months is at most 0.36. Given anticipated dropout based on prior studies, including between enrolment and randomization, we conservatively estimate we will need to recruit 200 participants to reach a sample size of *N* = 110 after 6 months.

### Recruitment {15}

Recruitment will occur nationally from multiple sources. To enroll *N* = 200 caregivers, the investigators will partner with a consulting firm specializing in Alzheimer’s disease research. This organization will develop a marketing strategy for materials such as flyers, in addition to their own proprietary in-person and online direct recruitment approaches. Researchers are also partnering with community-based organizations to share information via newsletters and existing social media accounts, such as through the California Caregiver Resource Centers (https://www.caregivercalifornia.org/). Researchers will also connect with direct service providers at these organizations to encourage them to share information with family caregivers with whom they work and provide a “warm hand off” to the study team [45]. Prior to enrolment, study team members will participate in a training informed by the *Network, Give first, Advocate for research, Give back, Evaluate, and Design and Develop* Model developed at the Rush University Alzheimer’s Disease Research Center [46–48]. This training will include a focus on recruiting caregivers who are severely underrepresented in current AD/ADRD research, including African American and Hispanic caregivers, as findings from the REACH II trial indicate culturally sensitive training may improve sample representativeness [49]. The recruitment approaches applied in this study will be dynamic. Ongoing monitoring of recruitment approaches, such as assessing enrolment following the use of a novel recruitment approach, will guide recruitment decisions. The researchers will recruit up to 16 cohorts of 12 to 24 caregivers each, such that randomized cohorts will be assigned to a group of at least *n* = 6 participants, but no groups will be larger than *n* = 12.

## Assignment of interventions: allocation

### Sequence generation {16a}

We will use stratified block randomization to assign participants to the intervention or control group at a 1:1 ratio. Consented participants will be randomized in variable block sizes once participants complete the baseline survey. Further, the study team will use stratification by race and ethnicity to prevent an unbalanced distribution of Hispanic and African Americans between the intervention and control conditions.

### Concealment mechanism {16b}

The randomization list will be generated in Stata 18 before the beginning of the trial. Only the PI and project manager can access the sequence file and REDCap project where randomization occurs. Block sizes will be known to the PI and project manager. Sequential randomization of cases as baseline surveys are completed, limited access to this schema, and pre-upload of the sequencing schema support concealment.

### Implementation {16c}

The PI will generate the allocation sequence in consultation with the statistician (BC) prior to enrolling any participants. This file will be imported into REDCap when study enrolment begins. When a new case is entered into the REDCap randomization project by the PI or project manager, the participant will be assigned to the intervention or control condition based on the order in which they completed the baseline survey. Group assignments will be communicated directly to interventionists by the PI or project manager to deliver the intervention. Despite providing a check of the randomization schema prior to enrolment, the statistician will be unable to determine participant allocation, since all data provided to the statistician will be de-identified and participant labels do not indicate the sequence in which they were enrolled.

## Assignment of interventions: blinding

### Who will be blinded {17a}

The study statistician, data analyst, and research assistants who are involved in post-randomization data collection will be blinded to the intervention assignment. Participants, the PI, and the project manager will not be blinded. To maintain blinding, the statistician and data analyst will receive de-identified data on which to run analyses, wherein allocation will be indicated using a binomial variable not defined in the study codebook. Data collectors will receive participant contact information from the project manager when survey collection is scheduled but will not be told about participant allocation. Participants will be informed that the data collector is unaware of which program they completed at the beginning of the call.

### Procedure for unblinding if needed {17b}

The design of this study is open label with only outcome assessors, the statistician and data analysts being blinded so unblinding will not occur.

## Data collection and management

### Plans for assessment and collection of outcomes {18a}

Research assistants will enter research data directly into REDCap while completing Zoom-administered surveys [50]. REDCap is a secure, HIPAA-compliant survey data collection platform. Research assistants will share slide decks with questions and response options listed as each item is asked to promote focus and remind participants of response options. All data collectors will complete training, which includes practicing administering at least one recorded practice survey.

### Plans to promote participant retention and complete follow-up {18b}

To support retention, caregivers will receive a welcome packet in the mail with their participant workbook. This welcome packet will include a letter from the investigator team thanking participants for their time and reminding them of the importance of trial participation. Participants will also receive a branded writing utensil and fridge magnet to remind them of their study participation with a message that reinforces the importance of their contribution. Prior research has found that similar motivational approaches can promote participant retention [51]. In addition, following best practices from prior trials to the extent that it is feasible, participants will work with consistent study staff and will have multiple options to schedule interview times [52, 53].

### Data management {19}

To check the accuracy of data entry, the project manager or PI will select 5% of surveys due for data collection for each cohort. Data quality checks will consist of video recording Zoom survey data collection and having a second study team member mark the accuracy of how response options were captured in a REDCap form. The program manager or PI will notify data collectors if their Zoom survey was selected for a quality check and will review data entry quality check reports monthly. If reports indicate a rate of data entry error at 5% or higher on any single survey, the person conducting data entry will undergo retraining. If this occurs a second time, the data collector will be removed from the study to prevent inaccurate data entry from affecting results.

### Confidentiality {27}

To preserve participant confidentiality, during the trial, participants identifying information will be stored in a separate REDCap folder from their outcome data. Each study participant will be identified using two unique IDs: a sequentially generated identifier used to track participant status in the trial and a second four-digit identifier generated using a random number sequence to label all study data. A study key to connect identifiable information to study data will be stored in an encrypted folder available only to the PI and project manager. When data collectors are assigned to collect follow-up survey data, they will be provided with the participant’s name, phone number, and a link to the designated study survey. All participant identifying information will be deleted at the end of the trial after a summary of study results is shared with participants.

### Plans for collection, laboratory evaluation, and storage of biological specimens for genetic or molecular analysis in this trial/future use {33}

Not applicable. The research team is not collecting biological specimens as a part of this study.

## Statistical methods

### Statistical methods for primary and secondary outcomes {20a}

#### Preliminary descriptive analyses

Prior to running models to test study hypotheses, the investigators will calculate means/standard deviations on continuous variables and frequencies/percentages for categorical variables for each survey (e.g., baseline) for both the intervention and control group participants. Analyses of primary and secondary outcomes will be stratified by race, ethnicity, and gender. We will visually analyze box-and-whisker plots to detect potential outliers.

#### Bivariate analyses

Next, three sets of paired *t*-test analyses will be conducted using primary and secondary outcome scores from baseline to the (1) post-intervention, (2) 3-month survey, and (3) 6-month survey for both the *LST* and active control group. We anticipate that *LST* participants will demonstrate statistically significant differences in mean self-efficacy and secondary outcomes for each *t*-test but that there will not be a significant difference between means for the active control group. We will also visually analyze spaghetti plots that chart each outcome variable.

#### Selecting covariates

Before running models to test study hypotheses, the investigators will conduct additional bivariate tests (e.g., Pearson chi-squared) to compare the characteristics of participants randomized into the intervention and active control groups to confirm that groups are balanced in terms of sample characteristics and possible confounders. Variables that demonstrate statistically significant differences in means or distribution at *α* = 0.10 between study arms will be included as model covariates. Important covariates such as age, gender, race, and ethnicity will be included in models, regardless of their balance between two groups.

#### Direct effects of the Learning Skills Together intervention

To test for intervention effects of *LST* compared to the control condition, we will apply generalized estimating equation (GEE) models with an identity link and an unstructured correlation matrix to data. The models will include group assignment (intervention), time, and interaction between the group and time. A multivariate Wald test will be performed to see whether there is any group × time interaction. Once this joint test yields a significant result (*α* = 0.05), we will test if each regression coefficient of the group × time interaction demonstrates a *p*-value of < 0.05. If the model coefficient shows a positive association with self-efficacy, this will support our hypothesis that participation in *LST* improves caregiver self-efficacy relative to participation in an active control group. These analyses will be repeated for the outcomes measuring self-efficacy with complex care tasks, as well as secondary outcomes of depressive symptomology and negative appraisal of BPSD.

#### Self-efficacy as a mediator of intervention effects

**Fig. 2:**
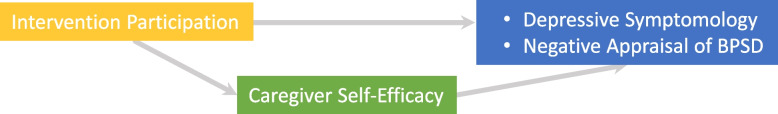
Mediation model to test self-efficacy as a mediator of secondary outcomes

To test self-efficacy as a mediator of secondary outcomes, we will adopt a structural equation modeling approach, which comprises two sequential models. In this analysis, the outcome will be the change score for depression or appraisal of BPSD, which is calculated by the score at 6 months minus the score at baseline. The mediator will be the change score for self-efficacy, tested using the Self-Efficacy Scale-8, calculated by 3 months minus the baseline score. Direct and indirect effects will be estimated using Baron’s and Kenny’s method [54] (Fig. 2).

#### Effect modification by race, ethnicity, and gender

To compare intervention effects on primary and secondary outcomes according to caregiver race, ethnicity, and gender, the investigators will first compare average change scores between study arms among African American vs. non-African American, non-white Hispanic vs. non-Hispanics, and female vs. male gender. Next, the researchers will include group × race/ethnicity/gender and group × time × race/ethnicity/gender effects in the GEE models to test effect modifications.

### Interim analyses {21b}

We will conduct one interim analysis when 50% of participants are randomized to evaluate intervention safety and futility. Results of the interim analyses will be made available to the investigators, SO, and NIA PO. Trial termination will only be considered in the following instances: (1) the trial is unable to recruit caregiver participants, (2) the investigators discover a change in average depression in the intervention group that (a) worsens following intervention and (b) worsens to a degree that is greater than any negative changes in the control group for this outcome, and/or (3) we discover reports of unexpected serious adverse events linked to participation in the *Learning Skills Together* intervention in more than 15% of caregivers.

### Methods for additional analyses, including by subgroup {20b}

#### Alternative theory testing with resourcefulness

In addition to primary analyses, the investigators will test resourcefulness as an alternative theory of intervention to explain hypothesized changes in depressive symptomology and negative appraisal of BPSD [32]. If mediation effects for resourcefulness are equal to or greater than those observed for self-efficacy, it will support the application of resourcefulness as an alternative or complementary mechanism of change.

#### Quality of care as an ancillary secondary outcome

Although the intervention is primarily focused on caregiver outcomes to support the delivery of complex care while supporting mental health, it is conceivable that participating in *Learning Skills Together* could affect care delivery. As such, quality of care, measured by the Task Management Strategy Index will be examined in the same manner as secondary outcomes [44].

#### Intervention dosage

Lastly, the investigators will conduct secondary analyses with a subsample of participants who participated in at least four of the six sessions for the assigned study arm. If a high level of non-adherence to the intervention is observed, it may weaken observed intervention effects due to limited intervention exposure. In this case, secondary analyses could help disentangle intervention efficacy from potential feasibility issues. Though failure to observe intervention effects in primary analyses would still indicate a lack of efficacy, secondary analyses could help inform the next steps in modifying and testing *Learning Skills Together*.

### Methods in analysis to handle protocol non-adherence and any statistical methods to handle missing data {20c}

Non-complete cases, due to situations such as changes in eligibility between survey waves (e.g., care recipient deceased) and skipped surveys, will be treated using an intent-to-treat analysis. This means that all data from participants who completed a baseline survey and were randomized will be analyzed as members of the group into which they were randomized. We will conduct bivariate comparisons between study completers and non-completers to determine factors associated with dropping out, including group assignment. Missing data at the construct level will be handled using multiple imputations using chained equations (MICE). Imputed data sets will be used to fit the GEE models. Covariates that demonstrate a bivariate association with missingness will be included as auxiliary variables to inform imputations. The results with imputation will be summarized using Rubin’s rules and compared with the GEE models using complete cases.

### Plans to give access to the full protocol, participant level-data, and statistical code {31c}

De-identified research data will be shared at the National Archive of Computerized Data on Aging (NACDA), an NIH-funded repository. Documentation, such as code names and original survey questions, and protocol documents, will also be uploaded to the repository in compliance with NACDA requirements (e.g., code names usable across software packages). We will also document meta-data (e.g., study title, investigator team). Data will be accessible to investigators working under an institution with a Federal Wide Assurance (FWA) and could be used for secondary study purposes. Sharing of analytic code will be provided at the discretion of the investigator team, based on the assessed qualification of the access requester, where “expert users” with an advanced academic degree or other relevant experience will be provided with code files. De-identified data will be made available as soon as possible but no later than within 1 year of the completion of the funded project period for the parent award or upon acceptance of the data for publication, or public disclosure of a submitted patent application, whichever is earlier.

## Oversight and monitoring

### Composition of the coordinating center and trial steering committee {5d}

The PI and project manager are at the core of the study coordinating center. They are responsible for hiring and coordinating staff, including undergraduate, graduate, and professional research assistants and associates, who will conduct routine activities such as data collection and day-to-day recruitment activities. This team will meet once per week, either in-person or remotely. The project manager will also coordinate nurse interventionists who will deliver the intervention and who are hired on a contract basis. Nurse interventionists will meet with the PI or project manager at least quarterly to identify challenges and coordinate coverage for upcoming sessions. The investigator team, responsible for monitoring study progress and providing input on how to respond to challenges, will meet monthly.

#### Composition of the data monitoring committee, its role, and reporting structure {21a}

Due to the low-risk nature of this trial, it was determined that a Data Safety Monitoring Board was not required. Instead, a qualified safety officer with relevant clinical and research experiences with family caregivers to persons living with AD/ADRD will review study safety concerns. The SO was approved by the sponsor but remains independent from the sponsor and has no competing interests. The SO will receive a report and meet twice per year with the PI to review study progress, including adverse events and the quality of data collection. The SO will report to the funder.

### Adverse event reporting and harms {22}

Adverse events are any untoward or unfavorable medical occurrence in a human study participant, including any abnormal symptom or disease, temporally associated with the participant’s involvement in the research, whether or not considered related to participation in the research. The only expected AEs with possibly deleterious outcomes for this study are risks of transient emotional upset experienced during research surveys and/or in processing their caregiving situation during the course of participating in the group-based online course.

Study team members with direct participant contact will complete role-play training to handle cases with risk of harm, such as suicidal ideation. Staff will receive a readily accessible flow chart/decision tree to provide quick guidance. In the case of suspected depression or suicidal ideation where the immediate risk of harm to self or others is not suspected, the study team will provide contact information for the 9–8-8 Suicide Crises Hotline and the Alzheimer’s Association 24/7 Helpline. If a participant affirms suicidal ideation on the PHQ-9 or has a PHQ-9 score of 15 or above, calculated automatically in REDCap survey forms, the threshold for moderately severe depression, we will notify participants of their elevated score and recommend consulting with their healthcare provider. Cases where we suspect elder mistreatment or neglect will be reported to the Adult Protective Services (APS) per Ohio’s mandatory reporting laws. The study team will undergo a learning session on APS referral during training before interacting with participants. When emergency scenarios arise, their handling will be discussed with the investigator team and SO to ensure compliance with emergency procedures. We will work with the SO and the participant on a case-by-case basis to determine whether the participant should be removed from the study due to severe depression or suicidal ideation.

Any unanticipated serious adverse event (SAE; e.g., death) related to the intervention will be reported to the NIA Program Officer, IRB, and the SO within 48 h of the study team’s knowledge of the SAE. Again, we do not anticipate any SAEs, as this is a low-risk study. Any unanticipated problem (e.g., a lapse in adherence to study procedures) involving risks to study participants or others that are related or possibly related to study participation will be reported to the NIA Program Officer within 48 h of the study team’s knowledge of the event. Self-monitoring, SO reports, and interim analyses, described in other sections, will help the study team to identify unanticipated problems that may occur. The study will provide the NIA Program Officer and the Safety Officer with quarterly reports of any SAE occurring during the study. All related or possibly related SAEs will be reported to the responsible IRB, as well as to the NIA Program Officer and the study’s SO at least annually.

### Frequency and plans for auditing trial conduct {23}

In addition to fidelity monitoring procedures described in 11c, data quality checks described in 19b, and SO reports described in section 21a, the research team will train all new staff to prevent non-adherence to study protocols, including rehearsal of all study procedures and regular team meetings to discuss unanticipated challenges among the study team. Prior to enrolling the first participant, the research team will conduct an internal audit of all procedures and materials. The PI will further internally audit records for completion and team adherence to protocols at least quarterly. The study team will also, following NIA requirements, upload study enrolment reports to the Clinical Research Operations & Management System (CROMS) system. Enrollment reports include characteristics for those screened and enrolled in the study and their status.

### Plans for communicating important protocol amendments to relevant parties (e.g., trial participants, ethical committees) {25}

Protocol amendments will be submitted to the responsible IRB before their implementation. Modifications that affect the risks of participation and trial experiences will be communicated to study participants by phone and/or email, including reconsenting of current and past participants if required by the IRB. Modifications will be reported to the SO at the following twice-yearly meeting to discuss safety monitoring. Changes to the study protocol following the publication of this manuscript will be specified in publications that report the results of the study objectives described in this submission.

## Dissemination plans {31a}

Our dissemination strategy employs multiple channels to reach professional and public audiences. To reach researchers and healthcare providers, findings will be presented in peer-reviewed journals, at academic conferences (e.g., Gerontological Society of America Annual Scientific Meeting), and conferences with a practice-based audience (e.g., American Society on Aging). Participants will also be provided with a written summary of findings in layperson language and invited to a webinar presentation of trial findings. If the trial demonstrated that *Learning Skills Together* is an evidence-based intervention, the investigators will also apply to have the intervention featured the Benjamin Rose Institute on Aging's *Best Practices in Caregiving* platform to drive broader adoption.

## Discussion

Findings from this study will help to support family caregivers of persons living with AD/ADRD who provide complex care, a type of caregiving that an increasing number of family members are providing to persons living with AD/ADRD and often other chronic conditions [4]. For individual caregivers, if *Learning Skills Together* demonstrates efficacy, it will provide another avenue to help families mitigate poor mental health associated with care stressors, such as the management of the BPSD that can make delivery of complex care challenging [4, 12]. Perhaps more importantly, identifying evidence-based approaches to help caregivers feel more prepared when providing complex care is highly relevant to the many caregivers who report feeling underprepared to provide complex care and worried about making a mistake [55]. At a societal level, developing evidence-based responses to better support family caregivers is more pressing than ever given the projected growth in the number of families affected by AD/ADRD. Current estimates project an increase in spending on long-term supports and services for persons living with AD/ADRD from US $345 billion in 2023 to nearly US $1 trillion by 2050 [1]. Preparation of family members may curb some of these costs, such as by delaying nursing home placement. Consequently, multiple payors in the USA are expanding payment options to support better access to interventions to support caregiver education and training, including the Department of Veteran’s Affairs and the Centers for Medicare and Medicaid Services [56]. Future research will aim to understand the implementation of the *Learning Skills Together* program in health and social care settings to determine the need for program modifications, appropriateness in various delivery settings, and facilitator training needs.

## Trial status

Study recruitment began on January 30, 2024. The current protocol version 1 is up to date as of May 24, 2024. Recruitment is anticipated to be completed by December 31, 2026.

## Data Availability

De-identified data generated from this study will be made available within the National Archive of Computerized Data on Aging (NACDA), which is an NIH-funded repository.

## Abbreviations

AD/ADRD: Alzheimer’s Disease and related dementias
BPSD: Behavioral and psychological symptoms of dementia
CROMS: Clinical Research Operations & Management System
GEE: Generalized estimating equations
LST: *Learning Skills Together*
RCT: Randomized controlled trial
SAE: Serious adverse events
SO: Safety officer

## References

[1] Alzheimer’s Association (2023). 2023 Alzheimer’s disease facts and figures. Alzheimers Dement.

[2] Hazzan AA, Dauenhauer J, Follansbee P, Hazzan JO, Allen K, Omobepade I (2022). Family caregiver quality of life and the care provided to older people living with dementia: qualitative analyses of caregiver interviews. BMC Geriatr.

[3] Reinhard S (2019). Home alone revisited: family caregivers providing complex care. Innov Aging.

[4] Lee M, Ryoo JH, Campbell C, Hollen PJ, Williams IC (2019). Exploring the challenges of medical/nursing tasks in home care experienced by caregivers of older adults with dementia: an integrative review. J Clin Nurs.

[5] Wolff JL, Spillman B (2014). Older adults receiving assistance with physician visits and prescribed medications and their family caregivers: prevalence, characteristics, and hours of care. J Gerontol B Psychol Sci Soc Sci.

[6] Wolff JL, Spillman BC, Freedman VA, Kasper JD (2016). A national profile of family and unpaid caregivers who assist older adults with health care activities. JAMA Intern Med.

[7] Burgdorf J, Arbaje A, Wolff JL (2020). Older adult factors associated with identified need for family caregiver assistance during home health care. Home Health Care Manag Pract.

[8] Riffin C, Van Ness PH, Wolff JL, Fried T (2017). Family and other unpaid caregivers and older adults with and without dementia and disability. J Am Geriatr Soc.

[9] Affoo RH, Foley N, Rosenbek J, Kevin Shoemaker J, Martin RE (2013). Swallowing dysfunction and autonomic nervous system dysfunction in Alzheimer’s disease: a scoping review of the evidence. J Am Geriatr Soc.

[10] Lagana V, Bruno F, Altomari N, Bruni G, Smirne N, Curcio S (2022). Neuropsychiatric or Behavioral and Psychological Symptoms of Dementia (BPSD): focus on prevalence and natural history in Alzheimer’s disease and frontotemporal dementia. Front Neurol.

[11] Fauth EB, Femia EE, Zarit SH (2016). Resistiveness to care during assistance with activities of daily living in non-institutionalized persons with dementia: associations with informal caregivers’ stress and well-being. Aging Ment Health.

[12] Pinyopornpanish K, Soontornpun A, Wongpakaran T, Wongpakaran N, Tanprawate S, Pinyopornpanish K (2022). Impact of behavioral and psychological symptoms of Alzheimer’s disease on caregiver outcomes. Sci Rep.

[13] Levine C, Halper D, Peist A, Gould DA (2010). Bridging troubled waters: family caregivers, transitions, and long-term care. Health Aff.

[14] Reinhard S, Caldera S, Houser A, Choula R. Valuing the invaluable: 2023 update. Washington, D.C.: AARP Public Policy Institute; 2023. https://www.aarp.org/content/dam/aarp/ppi/2023/3/valuing-the-invaluable-2023-update.doi.10.26419-2Fppi.00082.006.pdf. Accessed 21 May 2024.

[15] Burgdorf J, Roth DL, Riffin C, Wolff JL (2019). Factors associated with receipt of training among caregivers of older adults. JAMA Intern Med.

[16] National Alliance for Caregiving & Alzheimer’s Association. Dementia Caregiving in the U.S.; 2017. https://ogg.osu.edu/media/documents/sage/DementiaCaregivingFINAL_WEB.pdf. Accessed 21 May 2024.

[17] Cheng S-T, Li K-K, Losada A, Zhang F, Au A, Thompson LW (2020). The effectiveness of nonpharmacological interventions for informal dementia caregivers: an updated systematic review and meta-analysis. Psychol Aging.

[18] Sörensen S, Pinquart M, Duberstein P (2002). How effective are interventions with caregivers? An updated meta-analysis. Gerontologist.

[19] Bandura A (1997). Self-efficacy: the exercise of control.

[20] Crellin NE, Orrell M, McDermott O, Charlesworth G (2014). Self-efficacy and health-related quality of life in family carers of people with dementia: a systematic review. Aging Ment Health.

[21] George NR, Steffen A (2014). Physical and mental health correlates of self-efficacy in dementia family caregivers. J Women Aging.

[22] Khan TS, Hirschman KB, McHugh MD, Naylor MD (2021). Self-efficacy of family caregivers of older adults with cognitive impairment: a concept analysis. Nurs Forum.

[23] Gallagher D, Ni Mhaolain A, Crosby L, Ryan D, Lacey L, Coen RF (2011). Self-efficacy for managing dementia may protect against burden and depression in Alzheimer’s caregivers. Aging Ment Health.

[24] Grano C, Lucidi F, Violani C (2017). The relationship between caregiving self-efficacy and depressive symptoms in family caregivers of patients with Alzheimer disease: a longitudinal study. Int Psychogeriatr.

[25] Zhang S, Guo Q, Edwards H, Yates P, Li C (2014). Self-efficacy moderation and mediation roles on BPSD and social support influences on subjective caregiver burden in Chinese spouse caregivers of dementia patients. Int Psychogeriatr.

[26] Prado P, Norman RS, Vasquez L, Glassner A, Osuoha P, Meyer K (2022). An interprofessional skills workshop to teach family caregivers of people living with dementia to provide complex care. J Interprof Educ Pract.

[27] White CL, Barrera A, Turner S, Glassner A, Brackett J, Rivette S (2022). Family caregivers’ perceptions and experiences of participating in the learning skills together intervention to build self-efficacy for providing complex care. Geriatr Nurs.

[28] Meyer K, Glassner A, Norman R, James D, Sculley R, LealVasquez L (2022). Caregiver self-efficacy improves following complex care training: results from the Learning Skills Together pilot study. Geriatr Nurs.

[29] Bonds Johnson K, Higgins M, Epps F, Brewster GS, Alexander K, Hepburn K (2024). Tele-Savvy outcomes of non-Hispanic Black American and White caregivers. Gerontologist.

[30] Onken L, Kaskie B (2022). Implementation science at the National Institute on Aging: the principles of it. Public Policy Aging Rep.

[31] Zauszniewski JA, Burant CJ (2020). Resourcefulness as a mediator of the effects of dementia symptoms and caregiver reactions on caregiver mental health. Issues Ment Health Nurs.

[32] Zauszniewski JA (2016). Resourcefulness. West J Nurs Res.

[33] Reisberg B, Ferris SH, De Leon MJ, Crook T (1988). Global deterioration scale (GDS). Psychopharmacol Bull.

[34] Hepburn K, Nocera J, Higgins M, Epps F, Brewster GS, Lindauer A (2022). Results of a randomized trial testing the efficacy of Tele-Savvy, an online synchronous/asynchronous psychoeducation program for family caregivers of persons living with dementia. Gerontologist.

[35] Lorig K, Ritter PL, Laurent DD, Yank V (2019). Building better caregivers: a pragmatic 12-month trial of a community-based workshop for caregivers of cognitively impaired adults. J Appl Gerontol.

[36] Tang WK, Chan CY (2016). Effects of psychosocial interventions on self-efficacy of dementia caregivers: a literature review. Int J Geriatr Psychiatry.

[37] Ritter PL, Sheth K, Stewart AL, Gallagher-Thompson D, Lorig K (2020). Development and Evaluation of the 8-item Caregiver Self-Efficacy Scale (CSES-8). Gerontologist.

[38] Piggott CA, Zimmerman S, Reed D, Sloane PD (2017). Development and testing of a measure of caregiver confidence in medical sign/symptom management. Am J Alzheimers Dis Other Demen.

[39] Kroenke K, Spitzer RL, Williams JB (2001). The PHQ-9: validity of a brief depression severity measure. J Gen Intern Med.

[40] Arroll B, Goodyear-Smith F, Crengle S, Gunn J, Kerse N, Fishman T (2010). Validation of PHQ-2 and PHQ-9 to screen for major depression in the primary care population. Ann Fam Med.

[41] Teri L, Truax P, Logsdon R, Uomoto J, Zarit S, Vitaliano PP (1992). Assessment of behavioral problems in dementia: the revised memory and behavior problems checklist. Psychol Aging.

[42] Zauszniewski JA, Lai CY, Tithiphontumrong S (2006). Development and testing of the resourcefulness scale for older adults. J Nurs Meas.

[43] McClendon MJ, Smyth KA (2013). Quality of informal care for persons with dementia: dimensions and correlates. Aging Ment Health.

[44] Gitlin LN, Winter L, Dennis MP, Corcoran M, Schinfeld S, Hauck WW (2002). Strategies used by families to simplify tasks for individuals with Alzheimer's disease and related disorders: psychometric analysis of the Task Management Strategy Index (TMSI). Gerontologist.

[45] Epps F, Brewster G, Phillips JS, Nash R, Shah RC, Hepburn K (2022). Using a warm hand-off approach to enroll African American caregivers in a multi-site clinical trial: the handshake protocol. J Appl Gerontol.

[46] Gilmore-Bykovskyi A, Croff R, Glover CM, Jackson JD, Resendez J, Perez A (2022). Traversing the aging research and health equity divide: toward intersectional frameworks of research justice and participation. Gerontologist.

[47] Denny A, Streitz M, Stock K, Balls-Berry JE, Barnes LL, Byrd GS (2020). Perspective on the “African American participation in Alzheimer disease research: effective strategies” workshop, 2018. Alzheimers Dement.

[48] Marquez DX, Jaldin MA, Negrete M, Whitt-Glover MC, Glover CM (2023). Physical activity and health equity for middle-aged and older adults. Kinesiol Rev.

[49] Belle SH, Burgio L, Burns R, Coon D, Czaja SJ, Gallagher-Thompson D (2006). Enhancing the quality of life of dementia caregivers from different ethnic or racial groups: a randomized, controlled trial. Ann Intern Med.

[50] Harris PA, Taylor R, Thielke R, Payne J, Gonzalez N, Conde JG (2009). Research electronic data capture (REDCap)–a metadata-driven methodology and workflow process for providing translational research informatics support. J Biomed Inform.

[51] Jake-Schoffman DE, Brown SD, Baiocchi M, Bibeau JL, Daubenmier J, Ferrara A (2021). Methods-motivational interviewing approach for enhanced retention and attendance. Am J Prev Med.

[52] Teague S, Youssef GJ, Macdonald JA, Sciberras E, Shatte A, Fuller-Tyszkiewicz M (2018). Retention strategies in longitudinal cohort studies: a systematic review and meta-analysis. BMC Med Res Methodol.

[53] Forsat ND, Palmowski A, Palmowski Y, Boers M, Buttgereit F (2020). Recruitment and retention of older people in clinical research: a systematic literature review. J Am Geriatr Soc.

[54] Baron RM, Kenny DA (1986). The moderator-mediator variable distinction in social psychological research: conceptual, strategic, and statistical considerations. J Pers Soc Psychol.

[55] Reinhard S, Young H, Levine C, Kelly K, Choula R, Accius J. Home alone revisited: family caregivers providing complex care. AARP Public Policy Institute; 2019. https://www.aarp.org/content/dam/aarp/ppi/2019/04/home-alone-revisited-family-caregivers-providing-complex-care.pdf. Accessed 21 May 2024.

[56] Reyes AM, Thunell J, Zissimopoulos J (2021). Addressing the diverse needs of unpaid caregivers through new health-care policy opportunities. Public Policy Aging Rep.

